# Glycerol Valorization towards a Benzoxazine Derivative through a Milling and Microwave Sequential Strategy

**DOI:** 10.3390/molecules27030632

**Published:** 2022-01-19

**Authors:** Miguel Ángel Torres-Pastor, Claudia Espro, Maurizio Selva, Alvise Perosa, Antonio A. Romero Reyes, Sameh M. Osman, Rafael Luque, Daily Rodríguez-Padrón

**Affiliations:** 1 Grupo FQM-383, Departamento de Química Orgánica, Universidad de Cordoba, 14014 Cordoba, Spain; m.a.torres1998@hotmail.es (M.Á.T.-P.); qo1rorea@uco.es (A.A.R.R.); 2 Dipartimento di Ingegneria, Università di Messina, 98100 Messina, Italy; 3 Dipartimento di Scienze Molecolari e Nanosistemi, UniversitàCa’ Foscari di Venezia, 30123 Venezia, Italy; selva@unive.it (M.S.); alvise@unive.it (A.P.); 4 Chemistry Department, College of Science, King Saud University, P.O. Box 2455, Riyadh 11451, Saudi Arabia; smahmoud@ksu.edu.sa; 5 Scientific Center for Molecular Design and Synthesis of Innovative Compounds for the Medical Industry, People’s Friendship University of Russia (RUDN University), 117198 Moscow, Russia

**Keywords:** milling, microwave irradiation, glycerol valorization, benzoxazine derivative

## Abstract

Glycerol and aminophenol intermolecular condensation has been investigated through a milling and microwave-assisted sequential strategy, towards the synthesis of a benzoxaxine derivative. Mechanochemical activation prior to the microwave-assisted process could improve the probability of contact between the reagents, and greatly favors the higher conversion of aminophenol. At the same time, following a mechanochemical–microwave sequential approach could tune the selectivity towards the formation of a benzoxazine derivative, which could find application in a wide range of biomedical areas.

## 1. Introduction

The current depletion of fossil fuels, together with the increase in energy consumption, as well as the global warming trends and their consequences on the environment and human safety, have promoted the development of more sustainable protocols, according to green chemistry principles [1,2,3]. In this sense, biorefineries have emerged as a highly promising option for the environmentally friendly preparation of chemicals, fuels, and materials [4,5,6,7,8,9]. For instance, biodiesel is a well-known biofuel obtained either from animal fats or vegetal oils, through a transesterification reaction under alkaline conditions [10,11]. Such well-known processes also give rise to the formation of glycerol as a subproduct. In order to fully ensure the sustainability and economic viability of the biodiesel production, the valorization of glycerol must be carried out [12].

In this regard, several strategies have been proposed to date, including the preparation of solketal [13], acrolein [14], cyclic acetals [15], glycidol [16], glycerol carbonate [17], diacetins [18], and mono- and polyesters/ethers [19], among others. Furthermore, the use of glycerol has been also reported for the synthesis of aryloxypropanediols with pharmaceutical activity, via reaction with several phenol derivatives, such as *ortho*-methoxyphenol, *ortho*-methylphenol, and *para*-chlorophenol, to name a few [20]. The desired products have been obtained in good yields; however, the reactions require long reaction times of between 12 and 28 h under conventional heating conditions, depending on the phenolic compound. The synthesis of aryloxypropanediols has been mainly reported by nucleophilic attack of phenols and glycidol, 1-chloroglycerol, or epichlorohydrin. The use of the aforementioned chemicals has several disadvantages, mainly related to their toxicity. Therefore, the use and valorization of glycerol, with the in situ formation of glycerol carbonate, constitute a green synthetic pathway. Moreover, reaction with other phenolic compounds, such as 2-aminophenol for the formation of benzoxazine derivatives, have scarcely been investigated. Importantly, benzoxazine heterocyclic products, which are non-steroidal progesterone receptor agonists, could find a broad range of applications in biomedicine, as antidepressant, antiangiogenic, antidiabetic, hypolipidemic, and anticancer agents. In addition, such compounds possesses antiplatelet aggregation activity [21].

With the aim of investigating the reaction of glycerol with 2-aminophenol, and to decrease reaction times in comparison with conventional approaches reported for the reaction of glycerol with other phenolic compounds, microwave technologies could be a highly valuable option. It is worth remarking that microwave technologies could improve the heat transfer of a process, as well as reduce reaction times and give rise to novel chemical behaviors that are different from those observed under conventional conditions [22,23].

In addition to microwave technologies, the use of mechanochemistry for organic synthesis has sparked the attention of the scientific community in recent years [24,25,26]. Even though mechanically induced reactions have been carried out since the 4th century BC, the number of publications on mechanochemistry only started increasing approximately 20 years ago. Thus far, mechanochemistry has been broadly employed for the synthesis of advanced nanomaterials [27], for the extraction of nutraceuticals, and, even more recently, for organic synthesis, including the preparation of active pharmaceutical ingredients (APIs). Very interesting studies have focused on the employment of this methodology for Suzuki–Miyaura cross-coupling reactions [28], the synthesis of peptides [29], and even of DNA fragments [30], to name some examples. Remarkably, mechanochemistry is a highly sustainable, simple, and reproducible approach [31].

**Figure 1:**
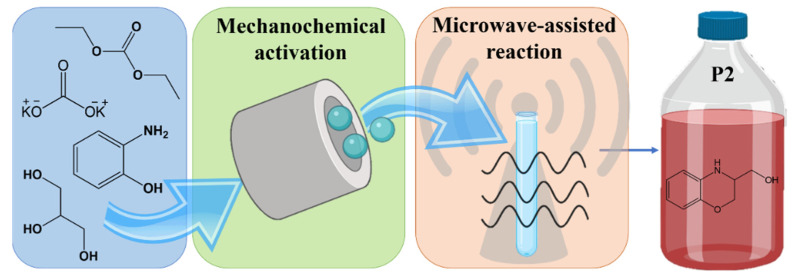
Illustrative representation of the reaction of 2-aminophenol with glycerol through a combined mechanochemical and microwave-assisted approach.

A sequential mechanochemical and microwave-assisted strategy has been proposed for the valorization of glycerol through reaction with 2-aminophenol, under solvent-free conditions (Figure 1). For comparison, several strategies have been considered, including a mechanochemically assisted approach, a microwave-assisted strategy, and a sequential mechanochemically activated and microwave-assisted methodology.

## 2. Results and Discussion

As a first approximation, the suitability of mechanochemistry for the condensation reaction of glycerol and 2-aminophenol was studied. A set of experiments was performed considering glycerol equivalents, milling time, and speed. However, low to negligible conversion values were achieved (Table 1, Entry 1), even when carrying out the reactions at a relatively high speed and over a prolonged time (Table 1, Entry 2, Table S1 Entry 10–13). These experiments indicated that, for the studied reaction and under the employed conditions, mechanochemical methods did not promote progress of the reaction, but, most probably, only the homogenization of the reaction mixture improved the probability of contact between the reagents. In addition, the influence of liquid-assisted grinding was explored by performing the reaction after adding 1 mL of acetone as a solvent (Table S1, Entry 14–17), also resulting in no significant improvement.

**Scheme 1:**
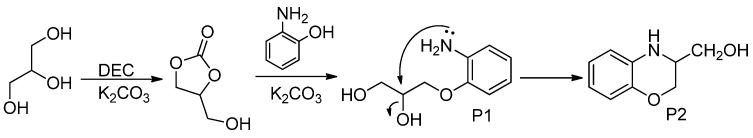
Schematic representation of the reaction of 2-aminophenol and glycerol.

Considering these results, we moved on to the use of microwave irradiation. By performing the reaction by employing K_2_CO_3_, under microwave irradiation, the formation of two products was observed, named as P1 and P2 [(3,4-dihydro-2*H*-benzo[b][1,4]oxazin-3-yl)methanol], in Scheme 1. The influence of glycerol equivalents on the progress of the reaction was investigated. The experiments were performed under microwave irradiation at 110 °C for 1 h. Microwave experiments displayed more promising results in comparison with the mechanochemically assisted approach. Preliminary results suggested that, by using 1 equivalent of glycerol, better results in terms of conversions were obtained, in comparison with higher glycerol equivalents. Furthermore, the influence of the catalytic system was investigated by performing the reaction in the absence of K_2_CO_3_. Such experiments showed a negligible conversion (<5%), confirming the important role of the employed base. In this sense, the use of potassium carbonate could promote the carbonatation reaction between glycerol and dialkyl carbonates (in this case diethyl carbonate) and, furthermore, promote nucleophilic attack of the phenolic compound in the cyclic carbonate.

Moreover, the effect of time and temperature on the microwave-assisted reaction was also explored. For this purpose, several experiments were carried out considering a higher temperature (150 °C) and using various reaction times (30, 60 and 120 min) (Table 1). By performing the reaction at 110 °C, it was observed that prolonged reaction times had a negligible effect on the 2-aminophenol conversion (regardless of the reaction time, the conversion was ca. 10 ± 2%), while it drastically affected products selectivity (Table 1, Entry 3–5). Such results suggest that the formation of P1 from glycerol and 2-aminophenol is a more challenging step in comparison with the formation of P2 from P1. In addition, the cyclization reaction is favored, with higher reaction times up to 60 min (Table 1, Entry 4). Nonetheless, at higher reaction times (120 min) (Table 1, Entry 5), a reverse hydrolysis reaction could most likely take place [32], promoting a higher selectivity towards P1. In addition, by carrying out the reaction at 150 °C (Table 1, Entry 6), the conversion increased from ca. 10% to 25%; however, selectivity towards P2 decreased considerably. Finally, the use of a higher speed during milling, in the sequential strategy, led to an increase in conversion to 46% and 57%, by using 600 rpm and 1000 rpm, respectively. Nonetheless, lower selectivities (Table S3, Exp 27–28) and, therefore, also lower yields (12% and 4% for the experiments performed at 600 rpm and 1000 rpm) of P2 were found in both cases.

Motivated by recent studies, where the combination of milling and microwave irradiation effectively favored the progress of reactions [33,34], a new set of experiments was designed by combining mechanochemical activation and microwave irradiation approaches (Table 1, Entry 8). Indeed, in addition to the energy input required in a chemical reaction, under solvent-free conditions, the mass-transport effect needs to be considered as a limiting factor [35,36]. In this regard, it is well-known that the kinetic energy produced during the milling gives rise to the fracture, abrasion, and refinement of the system microstructure. Consequently, the probability of contact between reagents could be enhanced and hence, mass transport limitations can be overcome [37,38]. By combining mechanochemical and microwave-assisted strategies, employing K_2_CO_3_ as catalytic system, a clear enhancement in the conversion and selectivity towards P2 was observed. Quantification analyses, according to GC results, revealed that the combined strategy (through mechanochemical activation and microwave irradiation) achieved the most promising results for the formation of P2, with conversion values of 38% and an outstanding selectivity of 93%. For comparison, the combination of milling and conventional heating was also investigated (Table 1, Entry 7, also for 1 h of reaction, to compare with the experiments under microwave irradiation); however, only conversions lower than 5% were achieved.

To compare the viability of the reaction for other phenolic derivatives, the reaction was carried out using phenol instead of 2-aminophenol (glycerol: phenol ratio: 1:1). In this case, the results were more promising, resulting in a complete conversion towards the desired product, which was identified using GC-MS (Figure S1). It can be stated that the obtained fragmentation pattern agrees with the expected compound, demonstrating that this methodology proceeds satisfactorily for the selective formation of the ariloxypropanodiol product.

Importantly, the results of the reactions under microwave irradiation at higher temperatures and for longer times confirmed that the reaction methodology, following a prior mechanochemical activation, certainly resulted in a better yield in the formation of the cyclic enamine, which is also most likely economically feasible. It has been reported that the ball milling processes required less energy in comparison with microwave irradiation, due to the lower efficiency of the transformation of electrical into microwave energy [39,40]. It is true that, herein, the designed strategy was based on a combination of milling and microwave energy, and that such an approach could be highly competitive, in terms of energy efficiency, in comparison with conventional heating strategies previously reported for these kinds of reactions with other phenolic compounds as long reaction times of up to 28 h have previously been required. Herein, for comparison, the reaction of glycerol and 2-aminophenol was performed in a parallel reaction station tube. The mixture was heated to 110 °C, achieving full conversion (>99%) after 46 h under stirring (a conversion of 38% was reached after 38 h). Such long reaction times have a direct negative impact on the energy and cost-efficiency of the process. An estimation in this regard has been included in the Supporting Information file. Atom economy and E-factor values were calculated, resulting in 82% and 2.2, respectively. In addition, and importantly, only the formation of P1 was observed, with no presence of P2, confirming the suitability of the milling–microwave sequential pathway for the selective synthesis of benzoxazine-derivatives.

The obtained MS spectrum (Figure S2) for the P1 product exhibited a molecular ion peak at *m*/*z* 181. In this case, it is worth noting that the lability of the hydrogen atoms in the OH groups could give rise to the appearance of molecular ions at *m*/*z* [M-H]^+^ and [M-2H]^+^; hence, the observed peak at *m*/*z* 181 could be most likely be associated with the successful formation of 2-aminoaryloxypropanediol (183 g/mol). Moreover, as observed in Figure S3A, the MS spectrum of the P2 peak displayed a molecular ion at *m*/*z* 165. Such *m*/*z* could most likely be associated with the loss of a water molecule (18 g/mol) from P1. This result could be interpreted as the intramolecular cyclization of P1, through a nucleophilic substitution of one of the hydroxyl groups by the amine group (Scheme 1). In order to corroborate the formation of P2, the pattern of the suspected molecule was acquired and analyzed via GC-MS (Figure S3B). Clear similarities were observed in the fragmentation pattern of both samples, which confirmed the formation of compound P2.

**Scheme 2:**
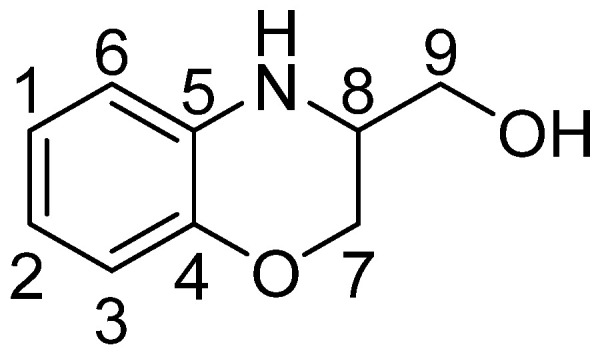
Schematic representation and numbering of the carbon atoms in (3,4-dihydro-2*H*-benzo[b][1,4]oxazin-3-yl)methanol.

Moreover, the P2 product was purified by column chromatography from the reaction media, employing ciclohexane-ethylacetate (7:3) as solvent system and silica as the stationary phase. The ^1^H and ^13^C NMR spectra of the pure product were acquired, confirming the formation of the proposed structure for the P2 product (Figures S4 and S5). The ^13^C NMR spectrum confirmed the presence of nine carbons, with the presence of six and three signals in the aromatic and aliphatic regions, respectively. ^13^C-NMR chemical shifts of 143.74 ppm and 133.42 ppm were associated with C(5) and C(4), respectively (Scheme 2). In addition, C(8) was assigned to the signal located at 51.23 ppm. Furthermore, ^1^H NMR displayed the presence of a multiplet signal at 3.43 ppm, integrating one proton, which was associated with the C(8)-H proton, strongly confirming the formation of the P2 product through the intramolecular cyclization of P1.

In addition, the FT-IR spectrum of P2 was acquired and is reported in Figure S6. The infrared spectrum showed a characteristic and wide band around 3369 cm^−1^, which can be attributed to the N–H stretching vibrations of the –NH groups, most likely together with an overlapped signal that is expected for the O-H group above 3500 cm^−1^. Moreover, signals located around 2938 cm^−1^ and 2878 cm^−1^ could be associated with C-H stretching vibrations and the O-C bond, respectively. The presence of a signal at ca. 1608 cm^−1^ could be attributed to N-H bend of the amine group, while the band located around 1500 cm^−1^ could be assigned to the C-C stretching vibration in the aromatic rings. Furthermore, the bands observed around 1436 cm^−1^ and 1360 cm^−1^ could be attributed to the C-H bend and the C-H rock of alkanes. Significantly, the peaks appearing at 1279 cm^−1^ and 1207 cm^−1^ can be attributed to the C–O stretching vibrations of alcohols and to the C-N stretching vibrations of amines, respectively, also supporting the formation of P2. Moreover, FT-IR analysis of P1 was also acquired (Figure S7), revealing a similar pattern, according to the expected similarities between the P1 and P2 structures. It is worth highlighting the clear difference observed for the bands above 3000 cm^−1^. In the case of P1, a wider signal could be observed, most likely due to the contribution of the two hydroxyl groups. In addition, it is well-known that, for primary amines (RNH_2_), two signals could be observed in the region above 3000 cm^−1^, namely the asymmetrical N–H stretch and the symmetrical N–H stretch, while only one signal related to NH group appears in the spectrum for secondary amines. Indeed, the obtained spectra are in accordance with the expected aforementioned results, since as could be observed for P1, there was a presence of two peaks within the wide band above 3000 cm^−1^.

## 3. Materials and Methods

Several strategies for the glycerol valorization have been considered, as described below.

### 3.1. Mechanochemically-Assisted Approach

A set of experiments was designed considering glycerol equivalents, milling time, and speed, as shown in Table S1. The reactions were carried out in ball mill reactors (Retsch PM100 ball mill model, Retsch Emax ball mill model) (Retsch, Düsseldorf, Germany), using a 125 mL reaction chamber and 60 g of 2 mm stainless steel balls.

### 3.2. Microwave-Assisted Approach

The reactions were performed employing the same reactants concentrations, varying the glycerol equivalents from 1 to 3. The experiments were performed under microwave irradiation in a CEM-Discover microwave reactor (CEM, Matthews, NC, USA), equipped with a PC-controlled interface, at 110 °C, for 1 h, employing a maximum power of 300 W and a maximum pressure of 240 bars (Table S2).

### 3.3. Combined Microwave and Mechanochemical-Assisted Approach

A set of experiments was designed, combining mechanochemical activation and microwave irradiation approaches (Table S3).

The conversion, selectivity, and yield were investigated using gas chromatography (GC) in an Agilent 6890N gas chromatograph (Agilent, Santa Clara, CA, USA) (60 mL min^−1^ N_2_ carrier flow, 20 psi column top head pressure) using a flame ionization detector (FID). An HP-5 capillary column (30 m × 0.32 mm × 0.25 mm) was employed. In addition, the collected liquid samples were analyzed using GC-MS using an Agilent 7820A GC/5977B High Efficiency Source (HES) MSD (Agilent, Santa Clara, CA, USA), in order to identify the obtained products. Moreover, the P2 product was purified by column chromatography, employing ciclohexane–ethylacetate (7:3) as the solvent system and silica as the stationary phase. Additionally, the ^1^H and ^13^C-NMR spectra were determined using a Bruker Avance III HD 400 WB equipped with a 4 mm CP/MAS probe, employing deuterated methanol.

Atom economy and E-factor values were calculated according to Equations (1) and (2).

(1) $$Atom\;economy=\frac{Molecular\;mass\;of\;product\;P2}{Molecular\;masses\;of\;reactants}\times 100\;\;$$

(2) $$E-factor=\frac{Mass\;of\;total\;waste}{Mass\;of\;product\;P2}$$

## 4. Conclusions

The use of novel technologies could lead to more sustainable scenarios for organic chemistry, giving rise to environmentally friendly industrial processes. In this work, the combination of milling, microwave technologies, and solvent-less conditions were explored for the valorization of glycerol towards a benzoxaxine derivative. Mechanochemical protocols could improve the contact between the reagents and favor mass-transport, while microwave irradiation leads to lower reaction times. This contribution could create new possibilities for the design of sustainable processes, helping the recognition of mechanical energy and its possible combination with other approaches for organic synthesis and for boosting products yields. As suggested by the estimation of energy usage, the present methodology could be competitive in comparison to conventional protocols, but more insightful and case-to-case research should be performed.

## Figures and Tables

**Table 1:** Performance of reactions of 2-aminophenol and glycerol, employing K_2_CO_3_. Quantification analysis was performed from GC-MS results.

Entry	Mechanochemical Conditions	Microwave Conditions/Conventional Heating	C * (%)	S ** (P2, %)	Y *** (%)
1	60 min, 350 rpm	-	<1	<1	<1
2	120 min, 350 rpm	-	<1	<1	<1
3		110 °C, 300 W, 30 min	10	32	3
4	-	110 °C, 300 W, 60 min	9	92	8
5	-	110 °C, 300 W, 120 min	12	45	5
6	-	150 °C, 300 W, 60 min	25	35	9
7 ^b^	60 min, 350 rpm	110 °C, 60 min	<5	<1	<1
8 ^a^	60 min, 350 rpm	110 °C, 300 W, 60 min	38	93	35

^a^ Conditions of the microwave-assisted reaction, ^b^ conditions of the reaction under conventional heating. * C: conversion (aminophenol); ** S(P2): selectivity towards (3,4-dihydro-2*H*-benzo[b][1,4]oxazin-3-yl)methanol, *** yield (P2).

## Data Availability

Not applicable.

## Supplementary Materials

The following supporting information can be downloaded, Figure S1: Comparison of MS-spectra (A) MS-spectrum of the product from the reaction employing phenol, instead of 2-aminophenol. (B) MS-spectrum reported in the NIST library; Figure S2: MS-spectrum of the obtained product by employing K_2_CO_3_, appearing at retention time of 11.8 min in the chromatograms. Figure S3: Comparison of MS-spectra of the product from the reaction, at retention time ca. 16 min, and the commercial pattern. (A) MS-spectrum of the obtained product by employing K_2_CO_3_, appearing at retention time of 16.7 min in the chromatograms. (B) MS-spectrum of the commercial pattern; Figure S4: ^1^H-NMR -spectrum of the purified product, appearing at retention time of 16.7 min in the chromatograms; Figure S5: ^13^C-NMR -spectrum of the purified product, appearing at retention time of 16.7 min in the chromatograms; Figure S6: FT-IR spectrum of the purified product P2, appearing at retention time of 16.7 min in the chromatograms; Figure S7: FT-IR spectrum of the purified product P1, appearing at retention time of 11.7 min in the chromatograms. Table S1: Set of experiments under ball milling conditions; Table S2: Set of experiments under microwave-assisted conditions; Table S3: Set of experiments under microwave and mechanochemical-assisted conditions.

## Sample Availability

Samples of the compounds are not available from the authors.
