# Supplementary material for: Glycerol Valorization towards a Benzoxazine Derivative through a Milling and Microwave Sequential Strategy
Source: Molecules. 2022 Jan 19;27(3):632. doi: 10.3390/molecules27030632 (PMC8838984; doi:10.3390/molecules27030632)
Supplement: Supplementary file 1 [file molecules-27-00632-s001.zip › molecules-1530409-supplementary.pdf]

Table S1. Set of experiments under ball milling conditions.

Exp.	Glycerol (eq.)	Solvent (1 mL)	Time (min)	Speed (rpm)	C (%)	S P2(%)
1	1	-	60	350	< 1	-
2	2	-	60	350	< 1	-
3	3	-	60	350	< 1	-
4	1	-	120	350	< 1	-
5	2	-	120	350	< 1	-
6	3	-	120	350	< 1	-
7	1	-	120	400	< 1	-
8	2	-	120	400	< 1	-
9	3	-	120	400	< 1	-
10	1	-	60	600	< 1	-
11	1	-	120	600	4	5
12	1	-	60	1000	6	6
13	1	-	120	1000	7	5
14	1	Acetone	60	350	< 1	-
15	1	Acetone	60	600	5	4
16	1	Acetone	60	1000	7	3
17*	1	-	60	350	< 1	-

Reaction conditions: 1 equivalent of 2-aminophenol (1 g), 1.4 equivalents of DEC (diethyl carbonate) (1.5 mL) and 0.1 equivalents of K_2CO_3. (0.12 g) * Employing phenol instead of 2-aminophenol.

Table S2. Set of experiments under microwave-assisted conditions.

Exp.	Glycerol (eq.)	t (min)	T (ºC)	Power	Max. Pressure	C (%)	S P2(%)
18	1	60	110	300	240	9	92
19	2	60	110	300	240	8	25
20	3	60	110	300	240	9	20
21	1	30	110	300	240	10	32
22	1	120	110	300	240	12	45
23	1	60	150	300	240	25	35
24*	1	60	110	300	240	> 99	98**

Reaction conditions: 1 equivalent of 2-aminophenol, (1 g), 1.4 equivalents of DEC (1.5 mL) and 0.1 equivalents of K_2CO_3. (0.12 g) * Employing phenol instead of 2-aminophenol. ** Selectivity to aryloxypropanodiol.

Table S3. Set of experiments under microwave and mechanochemical-assisted conditions.

Exp.	Mechanochemical activation	Microwave conditions				C (%)	S P2(%)
		t (min)	T (ºC)	Power	Max. Pressure		
23	30 min, 350 rpm	30	110	300	240	20	80
24	30 min, 350 rpm	60	110	300	240	30	92
25	60 min, 350 rpm	30	110	300	240	25	81
26	60 min, 350 rpm	60	110	300	240	38	93
27	60 min, 600 rpm	60	110	300	240	46	26
28	60 min, 1000 rpm	60	110	300	240	57	7

Reaction conditions: 1 equivalent of (2-aminophenol) (1 g), 1.4 equivalents of DEC (1.5 g) and 0.1 equivalents of K_2CO_3 (0.12 g) Glycerol (1 eq.).

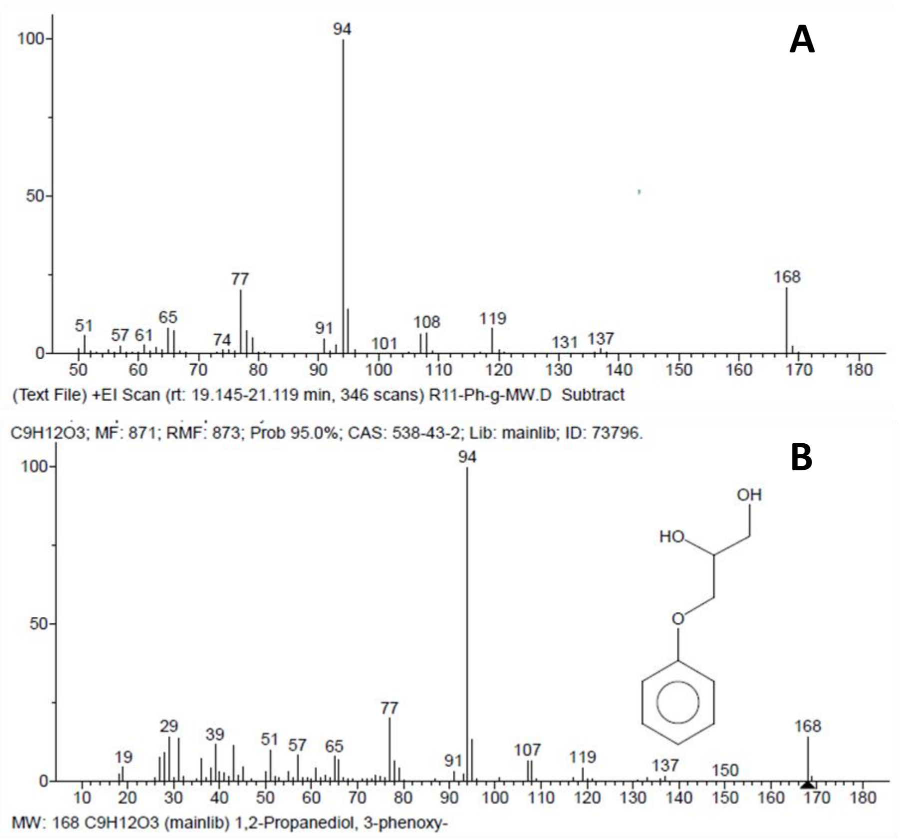

Figure S1. Comparison of MS-spectra A) MS-spectrum of the product from the reaction employing phenol, instead of 2-aminophenol. B) MS-spectrum reported in the NIST library.

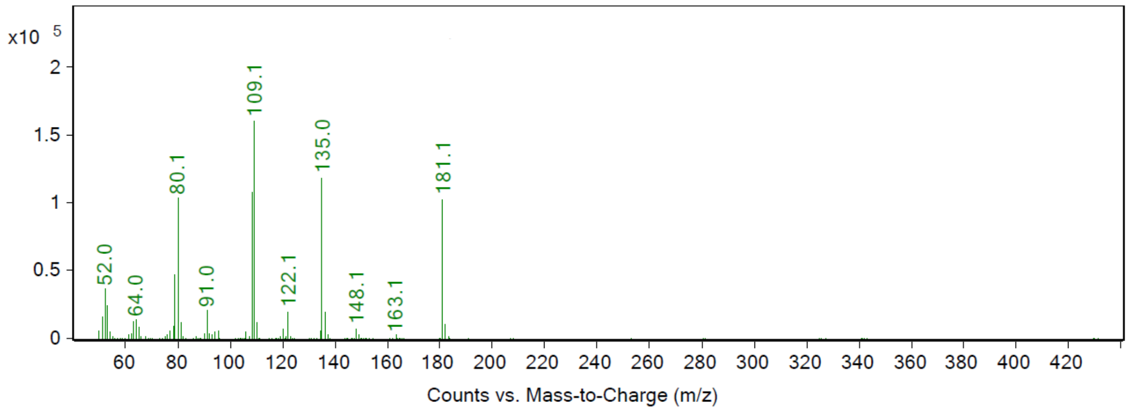

Figure S2. MS-spectrum of the obtained product by employing K_2CO_3, appearing at retention time of 11.8 min in the chromatograms.

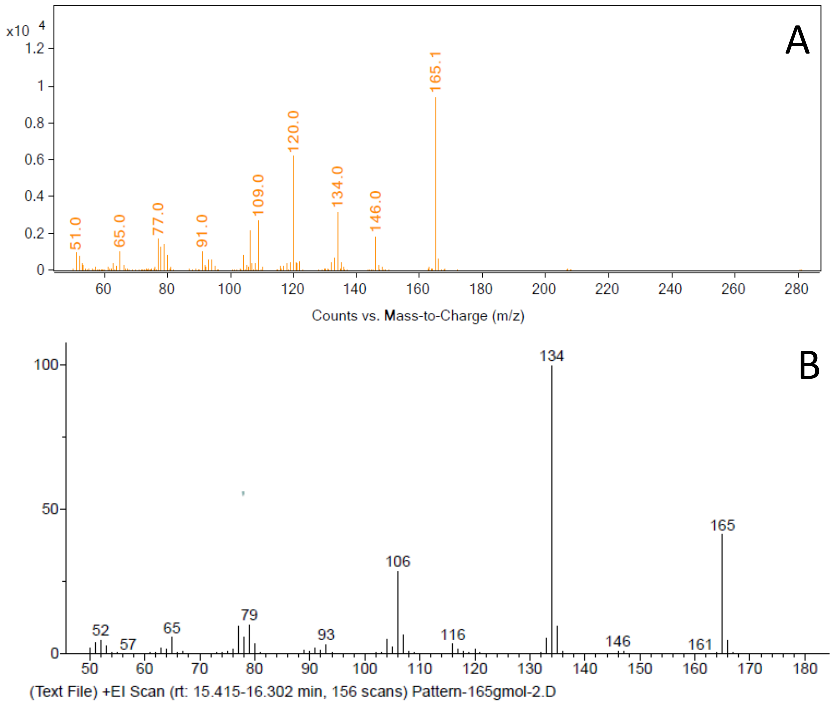

Figure S3. Comparison of MS-spectra of the product from the reaction, at retention time ca. 16 min, and the commercial pattern. A) MS-spectrum of the obtained product by employing K_2CO_3, appearing at retention time of 16.7 min in the chromatograms. B) MS-spectrum of the commercial pattern.

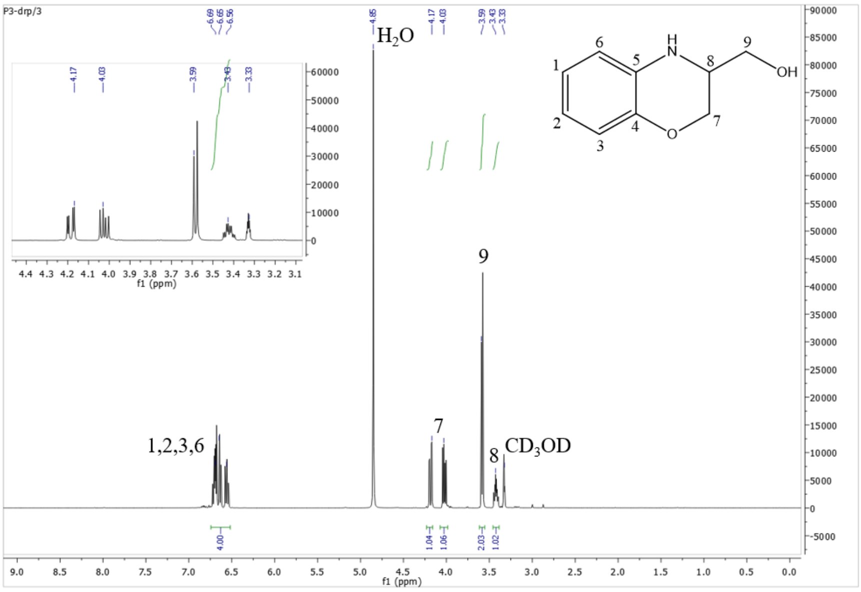

Figure S4. ^{1}H-NMR -spectrum of the purified product, appearing at retention time of 16.7 min in the chromatograms.

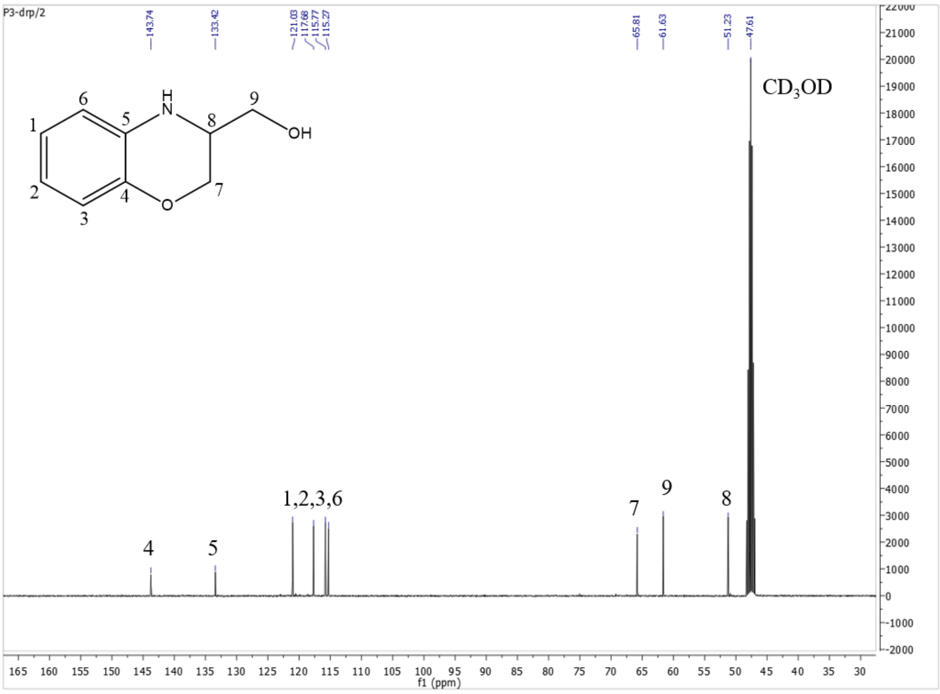

Figure S5. ^{13}C-NMR -spectrum of the purified product, appearing at retention time of 16.7 min in the chromatograms.

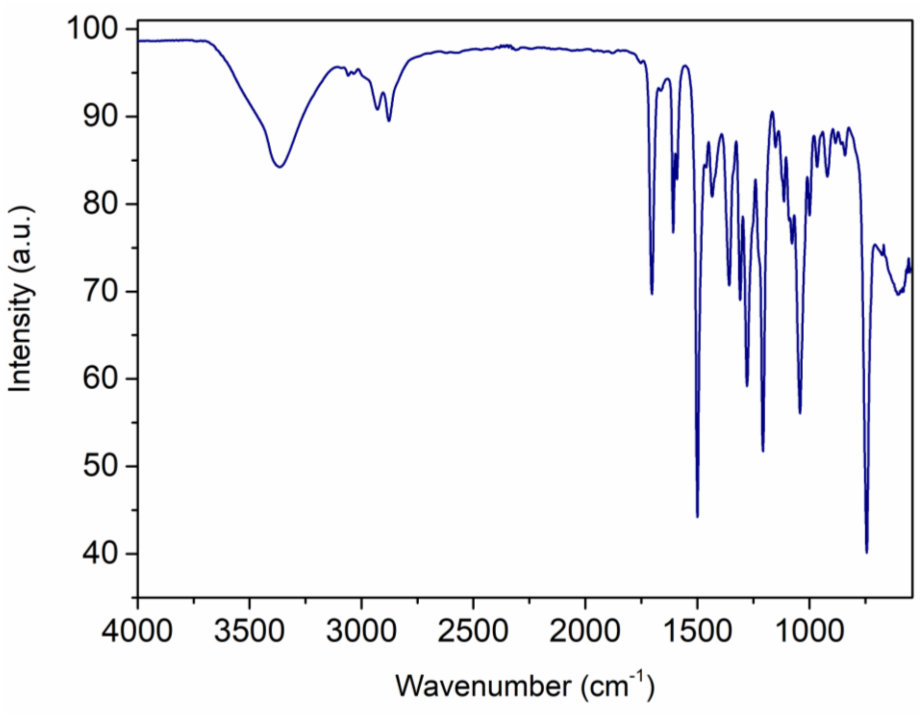

Figure S6. FT-IR spectrum of the purified product P2, appearing at retention time of 16.7 min in the chromatograms.

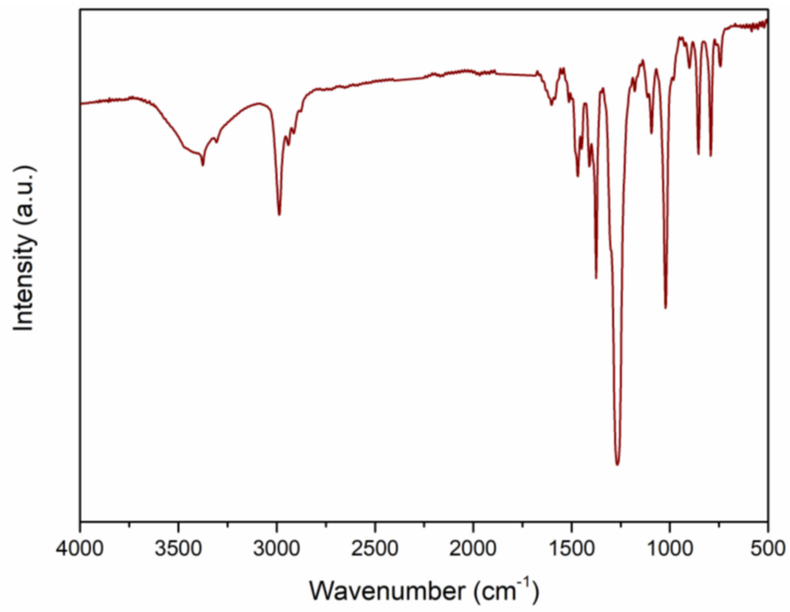

Figure S7. FT-IR spectrum of the purified product P1, appearing at retention time of 11.7 min in the chromatograms.

To perform the energy consumption assessment, as a rough measure, we approximated maximum power consumption, as stated in the Retsch PM (1250 W),[1] CEM microwave (1800 W)[2] and Parallel Reaction Station (800 W)[3] technical specifications. Taking this as our baseline, and considering the time employed in each case to achieve a conversion of 38% (obtained after 38 h for the conventional heating approach), we calculated the energy usage for both, the sequential mechanochemical-microwave assisted process and the conventional heating process, based on the following equation:

Energy usage(kW・h) = Power(kW) × Duration(h)

According to the aforementioned calculations energy usages of 3 KW/h and 30 KW/h was estimated for the sequential mechanochemical-microwave assisted process and for the conventional heating process, respectively. Even if further LCA analysis could be performed, this estimation revealed highly promising results in terms of energy efficiency.

[1]https://www.retsch.com/products/milling/ball-mills/planetary-ball-mill-pm-100/function-features/

[2]http://cem.com/media/contenttype/media/literature/516_Manual_MARS6_OperationManual_600284.pdf

[3]https://www.radleys.com/range/parallel-reaction-stations/
